# Recent Advances in Inhalation Therapy: An Overview

**DOI:** 10.3390/ph19020303

**Published:** 2026-02-11

**Authors:** Luca Casula, Francesco Lai

**Affiliations:** Department of Life and Environmental Sciences, University of Cagliari, S.P. Monserrato-Sestu km 0.700, Monserrato, 09042 Cagliari, Italy

## 1. Introduction

Inhalation has been employed for millennia as a means to deliver therapeutic agents directly to the respiratory tract, particularly for the local management of airway diseases such as asthma, bronchitis, chronic obstructive pulmonary disease (COPD), and cystic fibrosis [1,2,3]. Beyond its traditional use for localized treatment, the pulmonary route has also emerged as an attractive option for systemic drug administration, owing to several advantages over oral and parenteral delivery pathways [4]. This includes the lungs’ large surface area, thin epithelial barrier and rich vascular network, which enable rapid absorption and efficient delivery even for biologic drugs [5].

Despite these benefits, pulmonary drug administration is not exempt from limitations. Achieving reliable and efficient deposition of therapeutic agents within the bronchial or alveolar regions remains challenging due to the highly branched anatomy of the airways and the action of innate defense mechanisms, including mucociliary clearance, macrophage phagocytosis, proteolytic enzymes, and pulmonary surfactants [6,7].

The correct use of an inhaler device and the administration procedure is another factor that could hinder treatment effectiveness, as it can be more complex compared to oral administration. This could lead to variability in the administered dose, particularly in pediatric or older patients. For this reason, device usability, patient training, and intuitive design play a crucial role in minimizing errors and achieving consistent drug delivery across diverse patient populations [8,9]. Moreover, the efficacy and safety of inhaled therapies depend critically on the physicochemical properties of the formulation, and on its aerodynamic behavior once aerosolized and transported by the respiratory airflow [10]. Over recent years, extensive research efforts have been dedicated to designing new strategies to overcome sub-optimal drug deposition and impaired absorption.

Formulations intended for inhalation need to be carefully tailored to the specific device with which they will be administered (e.g., nebulizer, pressurized metered-dose inhaler (pMDI) or dry powder inhaler (DPI)), as they operate through different aerosolization principles, imposing unique requirements in terms of physicochemical properties, particle engineering, and dispersion behavior [11].

Among the physicochemical properties, drug solubility plays a pivotal role in the formulation development and the final efficacy of the therapy [12]. Several strategies have been employed to address solubility-related challenges, including the use of advanced carriers such as nanoparticles (NP), liposomes, and cyclodextrins, which can enhance dissolution, improve stability, and facilitate more efficient deposition within the respiratory tract [13,14,15].

Regarding particle engineering, a common strategy involves the use of polymeric excipients, which have gained increasing attention in pulmonary drug delivery. Both natural and synthetic polymers have been used to formulate aerosols, nebulizable systems, or dry powders, each offering specific advantages and limitations. A deep understanding of their structure, physicochemical properties, and deposition patterns is therefore essential for successful clinical translation [16].

Particularly, carbohydrate polymers such as chitosan, hyaluronic acid and mannitol have been used—beyond their biocompatibility and biodegradability—to refine the performance of spray-dried powders. These materials can impart favorable changes in surface morphology, reduce particle cohesiveness and enhance dispersibility, but also improve mucoadhesiveness and the drug residence time, to achieve long-acting inhaled dosage forms [17,18].

The advantages of nanocarriers and polymer/carbohydrate microparticles can be synergistically combined through nano-into-micro systems, in which nanocarriers are embedded within spray-dried or engineered microparticles. This strategy allows the retention of nanoscale benefits—such as enhanced solubility, protection of labile molecules, improved cellular uptake, and controlled release—while leveraging the superior aerodynamic properties and handling stability of micron-sized powders [19,20,21,22].

## 2. Contributions

In this Special Issue, these challenges and innovations have been explored through a collection of original research articles and reviews that address the most recent advances in inhalation therapy.

In **Contribution 1** [23], the authors provide a comprehensive review of nanoparticle-based strategies for pulmonary drug delivery, outlining key advantages, current challenges and future prospects in NP-based therapies. The article offers a critical overview of the main considerations in designing inhalable nanoparticles, emphasizing how drug-loading capacity, colloidal stability, and aerodynamic behavior collectively determine their clinical suitability. The discussion spans major nanoparticle classes—including lipid-based carriers, polymeric and protein nanoparticles, metallic and silica systems, quantum dots, exosome-mimetic structures, as well as nanocrystals and nanosuspensions—highlighting their distinct technological features. The review also provides some considerations on the use of the correct inhalation device in relation to these nanosystems.

**Contribution 2** [24] presents a representative research study that applies these nanotechnological concepts to a specific therapeutic scenario, offering experimental evidence on Ceftriaxone-Loaded Liposomal Nanoparticles. The formulations were prepared by a thin film hydration technique, leading to liposomal nanoparticles with an average diameter in the range of 90–536 nm and a spherical morphology that was retained even after freeze-drying. The samples showed a prolonged release over 24 h followed the Hixon–Crowell model, with CTX being transported through Fickian diffusion. Finally, the aerosolization studies—evaluated at a flow rate of 60 ± 5 L/min using a twin-stage impinger (TSI)—showed fine particle fraction (FPF) between 47 and 62%.

The role of polymers in particle engineering—previously introduced in the context of excipient functionality—is examined in depth in **Contribution 3** [25], where different polymer classes are critically discussed. This review outlines the advantages of natural and synthetic biopolymers in inhalation technologies, highlighting their tunable properties, biodegradability, and capacity to modulate release kinetics. Particular emphasis is placed on physiological barriers that limit pulmonary deposition and how polymer-based systems can mitigate them. The article then presents the major categories of biopolymeric microparticles, detailing preparation techniques and processing variables, and concludes with key considerations related to safety and stability.

**Contribution 4** [26] investigates the efficacy of a new device and its aerosol reproducibility for achieving site-specific microparticles deposition in the retropharynx. The study evaluates microparticle size and distribution using Low-Angle Laser Light Scattering across six APIs commonly used for rhinopharyngeal conditions and compares four nebulization devices. No significant differences emerged among the tested formulations; however, Rinubes showed markedly superior performance, generating an aerosol cloud with a significantly smaller MMD than the comparator devices. This finer aerosol is more suitable for retropharyngeal targeting. A retrospective analysis involving 74 patients additionally demonstrated improved clinical outcomes with nebulized Sobrerol, showing higher odds of resolving cough and nasal symptoms compared with standard therapy.

The functionality of mannitol as a key component within microparticle matrices is investigated in **Contribution 5** [27], with particular attention to its impact on particle morphology, dispersibility, and moisture stability. The study explores spray-dried inhalable microparticles containing meloxicam or its potassium salt, combined with poloxamer-188, mannitol, and leucine. The manufacturing process yielded spherical particles below 5 µm with partial amorphization. Dissolution testing showed rapid drug release, with more than 90% of both APIs dissolving within five minutes. Aerodynamic assessments demonstrated emitted fractions above 95%, fine particle fractions around 50%, and aerodynamic diameters near 4 µm. Spraytec analysis further confirmed their suitability for deep lung deposition, supporting the versatility of this excipient system for inhalable dry powders development.

Finally, **Contribution 6** [28] explores nano-to-microparticle engineering, focusing on the encapsulation of nanocrystals into respirable microparticles to improve handling, aerodynamic behavior, and dissolution performance upon deposition. Curcumin nanocrystals were produced via wet-ball milling in form of a nanosuspension stabilized with Poloxamer P188. The nanocrystals were subsequently incorporated into mannitol-based microparticles through spray drying, which modified particle morphology and flow properties compared to empty microparticles, while still ensuring optimal aerodynamic performance (MMAD < 5 µm). Dissolution and release studies in simulated lung fluids further evidenced the role of mannitol in modulating curcumin release profiles. Overall, the nanocrystal-loaded microparticles exhibited efficient aerosolization and controlled release, demonstrating the value of the nano-into-micro approach in combining nanoscale functionality with the handling and aerodynamic advantages of micronized powders for pulmonary drug delivery.

## 3. Conclusions

In conclusion, the contributions gathered in this Special Issue highlight the remarkable progress being made in the field of pulmonary drug delivery, where advances in particle engineering and device–formulation integration are converging to reshape therapeutic strategies for respiratory diseases. Collectively, the works presented here illustrate how innovative formulation approaches—from nanoscale systems to engineered microparticles and optimized aerosol performances—are driving the development of more efficient, stable and patient-oriented inhaled therapies. Continued research and cross-disciplinary collaboration will be essential to further refine these technologies and translate them into impactful clinical solutions.
